# Ginsenosides Rg1 and CK Control Temozolomide Resistance in Glioblastoma Cells by Modulating Cholesterol Efflux and Lipid Raft Distribution

**DOI:** 10.1155/2022/1897508

**Published:** 2022-10-10

**Authors:** Runze Qiu, Jingjing Zhang, Chun Ge, Yue Zhong, Suo Liu, Qingquan Li, Jianjun Zou, Hongwei Fan, Yingbin Li

**Affiliations:** ^1^Department of Clinical Pharmacology Lab, Nanjing First Hospital, Nanjing Medical University, Nanjing 210006, China; ^2^Department of Pharmacy, School of Basic Medicine and Clinical Pharmacy, China Pharmaceutical University, Nanjing 210009, China; ^3^Center of Drug Discovery, State Key Laboratory of Natural Medicines, China Pharmaceutical University, Nanjing 210009, China; ^4^Department of Neurosurgery, The Second Affiliated Hospital of Nanjing Medical University, Nanjing 210011, China

## Abstract

**Background:**

Cholesterol efflux and lipid raft redistribution contribute to attenuating temozolomide resistance of glioblastoma. Ginsenosides are demonstrated to modify cholesterol metabolism and lipid raft distribution, and the brain distribution and central nervous effects of whose isoforms Rb1, Rg1, Rg3, and CK have been identified. This study aimed to reveal the role of Rb1, Rg1, Rg3, and CK in the drug resistance of glioblastoma.

**Methods:**

The effects of ginsenosides on cholesterol metabolism in temozolomide-resistant U251 glioblastoma cells were evaluated by cholesterol content and efflux assay, confocal laser, qRT-PCR, and Western blot. The roles of cholesterol and ginsenosides in temozolomide resistance were studied by CCK-8, flow cytometry, and Western blot, and the mechanism of ginsenosides attenuating resistance was confirmed by inhibitors.

**Results:**

Cholesterol protected the survival of resistant U251 cells from temozolomide stress and upregulated multidrug resistance protein (MDR)1, which localizes in lipid rafts. Resistant cells tended to store cholesterol intracellularly, with limited cholesterol efflux and LXR*α* expression to maintain the distribution of lipid rafts. Ginsenosides Rb1, Rg1, Rg3, and CK reduced intracellular cholesterol and promoted cholesterol efflux in resistant cells, causing lipid rafts to accumulate in specific regions of the membrane. Rg1 and CK also upregulated LXR*α* expression and increased the cytotoxicity of temozolomide in the presence of cholesterol. We further found that cholesterol efflux induction, lipid raft redistribution, and temozolomide sensitization by Rg1 and CK were induced by stimulating LXR*α*.

**Conclusions:**

Ginsenosides Rg1 and CK controlled temozolomide resistance in glioblastoma cells by regulating cholesterol metabolism, which are potential synergists for temozolomide therapy.

## 1. Introduction

Glioblastoma (GBM) is the most frequent and most malignant (WHO IV) central nervous system (CNS) tumor. According to the latest statistics [1], the proportion was 54.4% among CNS tumor patients ≥20 years old, which rises with age. In the past 40 years, the treatment of GBM has not made significant progress, and the 5-year survival rate of patients has only increased from 4% to 7%. DNA alkylating agent temozolomide (TMZ) is the first-line drug for GBM, used in conjunction with radiotherapy and further adjuvant therapy after maximal safe surgical resection [2]. It has completed oral absorption, depressed plasma protein binding, metabolism not affected by liver and kidney status, and easy penetration of the blood-brain barrier (BBB) [3]. Nonetheless, drug resistance developed in about half of the patients [4]. They relapsed within 6 months of TMZ standard treatment and had to start a second-line treatment with deficient standards, higher toxicity, and less benefit [5]. O6-methylguanine-DNA methyltransferase (MGMT) and drug efflux transporters such as multidrug resistance protein 1 (MDR1, also known as ATP-binding cassette (ABC)B1 or P glycoprotein) and ABCG2, are still independent risk factors for TMZ resistance [6–8]. Strategies to address resistance need to be established.

**Figure 1:**
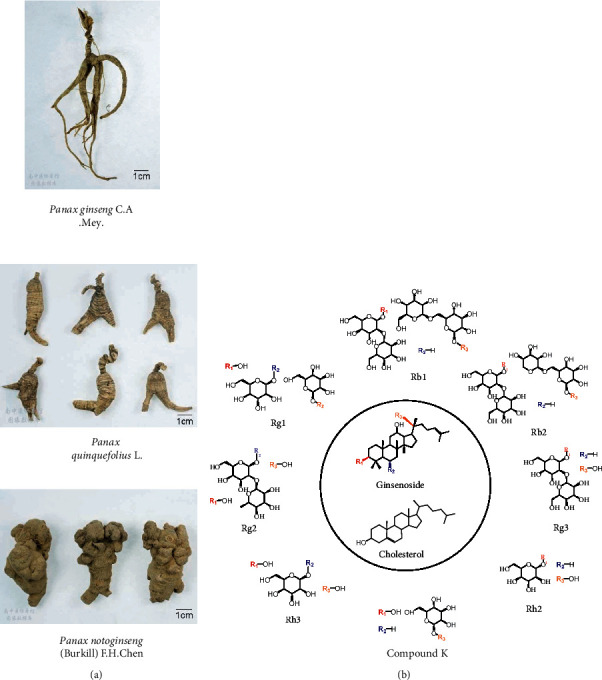
*Acanthaceae*, *Panax genus*, and ginsenosides. (a) The photographs of 3 representative Panax genus plants from the Chinese medicine specimen center of Nanjing University of Chinese Medicine (https://zybb.njucm.edu.cn/). (b) The structure of ginsenosides and cholesterol. The core steroid structure of ginsenosides is similar to cholesterol.

In traditional medicine, the herbs of the *Panax genus*, *Araliaceae*, including ginseng (*Panax ginseng* C.A. Mey.), American ginseng (*Panax quinquefolius* L.), and pseudo-ginseng (*Panax notoginseng* (Burkill) F.H. Chen), which can “nourish vitality” and “disperse stasis” [9], were used to combat the pathogenesis of glioma [10]. They exhibited therapeutic activities against tumors including GBM and have been commonly used in East Asia as adjuvant drugs [10, 11]. Ginsenosides containing numerous subtypes (Figure 1) are active ingredients with high content in *Panax genus* [11], and their saponin structure makes them amphipathic and BBB permeability, which have been confirmed by generally reported CNS effects. For instance, the oral pharmacokinetics of total ginsenosides indicated high concentrations of subtype Rg1 and compound K (CK) in brain tissues [12, 13]. Rb1, the precursor of CK, can enter the brain through glucose transporter member 1 (GLUT1) [14]. Moreover, Rg3 was identified to attenuate the TMZ resistance of GBM *in vivo* and *in vitro* [15].

As the most cholesterol-rich organ [16], the brain provides a unique microenvironment for GBM. GBM cells not only retain the ability of glial cells to synthesize cholesterol de novo but also increase cholesterol uptake mediated by low-density lipoprotein receptor (LDLR) [17]. Stimulating the liver X receptor (LXR) to promote ABCA1-mediated cholesterol efflux and degradation of LDLR induced GBM cell death [18–20]. It seems that the enormous demand for cholesterol from GBM cells is not just for the production of organelles. The increase in cholesterol and LDL levels promotes resistance of various tumors to chemotherapy, and it is also an independent risk factor for poor prognosis of GBM under standard TMZ treatment [21, 22]. Although cholesterol-induced drug resistance has not been confirmed in CNS tumors, elevated cholesterol in malignant ascites was proved to induce chemotherapy resistance by upregulating the drug efflux proteins ABCG1 and MDR1 [23]. MDR1 is located and stably expressed in lipid rafts composed of cholesterol, sphingolipid, and proteins on the plasma membrane [24]. When lipid rafts were induced to accumulate in specific parts of the membrane, MDR1 expression was confined, and tumor cells were less resistant [25]. Moreover, additionally added cholesterol restored the homogenous (broad and low-density) distribution of lipid rafts and upregulated MDR1 [25]. Conversely, lipid rafts were depleted when cholesterol was scavenged, and chemotherapy resistance was correspondingly diminished [26].

Both ginsenosides and cholesterol have steroid core structures (Figure 1), and ginsenosides can regulate cholesterol metabolism in tumor cells. Rg3 and CK hindered cholesterol synthesis by inhibiting *β*-hydroxy-*β*-methylglutaryl coenzyme A (HMG-CoA) [27, 28], and CK also promoted cholesterol efflux by stimulating LXR*α* [29, 30] to synergistically decrease intracellular cholesterol. Rh2 revealed lipid raft depleting activity [31, 32]. More direct evidence indicates that the ginsenoside derivative Rp1 decreased MDR1 activity by redistributing lipid rafts and attenuated the cholesterol-dependent resistance [25]. Consequently, ginsenosides are potential agents for the treatment of tumor resistance as regulators of cellular cholesterol metabolism. However, the differential roles of ginsenoside subtypes that govern cholesterol metabolism and drug resistance in CNS tumors remain undisclosed. In this study, we certified the role of cholesterol in TMZ resistance of glioblastoma U251 cells and investigated the effects of several ginsenosides with BBB penetration on TMZ resistance of U251 cells in the presence of cholesterol. We further studied the roles of ginsenosides on cholesterol metabolism and lipid raft distribution and the mechanisms controlling TMZ resistance. Our work suggests that ginsenosides Rg1 and CK are potential drugs to control TMZ resistance, providing references for adjuvant therapy of GBM.

## 2. Materials and Methods

### 2.1. Chinese Glioma Genome Atlas (CGGA) RNA-seq Analysis

From the CGGA database (https://www.cgga.org.cn/, China), which is a professional clinical transcriptome database of glioma, the mRNA-seq_693 dataset [33] was downloaded to attain the clinical information and sequencing data of samples. Among the 693 glioma patients, those who were known to be ≥18 years old, diagnosed with WHO grades III to IV, and treated by TMZ were screened. To eliminate the interference from the data of surviving patients with a short observation period, we excluded the surviving patients with less than 6 months of overall survival (OS). The mRNA-seq data were used to evaluate the correlation between OS, tumor tissue MGMT methylation status, fragments per kilobase transcriptome per million (FPKM) fragments of drug resistance gene *MDR1*, and FPKM of cholesterol metabolism genes including *LDLR*, *SREBF2*, *HMGCR*, *NR1H3*, *ABCA1,* and *ABCG1* via the Pearson correlation test.

### 2.2. Cell Culture, Resistance Induction, and Treatment

U251 human GBM cells (U251 MG, KeyGEN, ^#^KG050) were cultured in high-glucose DMEM (KeyGEN, ^#^KGM12800-500) containing penicillin, streptomycin, and fetal bovine serum (Gibco, ^#^30044333), with a constant ambient temperature of 37°C, saturated humidity, and 5% CO_2_. After stable growth, the TMZ-resistant U251 cells were induced by the dose escalation [34]. The escalating doses of TMZ (MCE, ^#^HY-17364) were 0.645, 1.29, 2.58, 5.16, 10.32, 20.64, 41.28, and 82.56 *μ*M. When the cells treated with the previous dose had grown to a confluency >50% and were stably passaged 2–3 times, the treatment was continued with the next dose. Induction of TMZ resistance was accomplished when cells were stably grown at 82.56 *μ*M TMZ. The Jiangsu KeyGEN BioTECH was commissioned to identify the genetic stability of the constructed TMZ-resistant cells by comparing the alleles on short tandem repeat (STR) and Amelogenin locus of the resistant cells with the data of parental U251 cells (Supplementary Figures S1 and Table S1). TMZ-resistant and wild-type U251 cells were treated with 0.78, 1.56, 3.13, 6.25, 12.5, 25, 25, 50, and 100 *μ*M TMZ with DMSO as a control, and IC_50_ and resistance index were calculated by Cell Counting Kit (CCK)-8 (KeyGEN, ^#^KGA317).

Cells were collected when rapidly grown to 60–80% density after passaging and treated with 5 *μ*g/ml cholesterol (dissolved in anhydrous ethanol, Solarbio, ^#^C8280) for 24 h depending on the grouping needs, and an equal volume of anhydrous ethanol was used as control. After the cells were washed, 200 *μ*M TMZ was added to continue treatment for 72 h, and an equal volume of DMSO was used as the control. Referring to the dose reported in GBM cells [15], 100 *μ*M ginsenosides Rb1 (Yuanye, ^#^B21050), Rg1 (Yuanye, ^#^B2105), Rg3 (Yuanye, ^#^B21059), and CK (Yuanye, ^#^B21045), and 100 nM LXR inhibitor GSK2033 (MCE, ^#^HY-108688) were used to treat cells alone or concurrently with TMZ for 72 h, with an equal volume of DMSO as the control.

### 2.3. Cell Proliferation and Cytotoxicity Assay

The cells were cultured in 96-well plates and treated according to the above protocol. CCK-8 reagent (KeyGEN, #KGA317) was added in plates 3 h before the end of administration, and the cells continued to incubate in the original culture environment. After 3 h, the plates were shaken and placed into the microplate reader (BioTek ELx800, USA), and the optical density (OD) value was read at 450 nm to calculate the inhibition rate. Inhibition rate = (1 − OD_Test well_/OD_Control well_) × 100%.

### 2.4. Flow Cytometry for Apoptosis and Cell Cycle Analysis

After 72 h of administration, cells were digested and collected with 0.25% EDTA-free trypsin (KeyGEN, ^#^KGM27250), washed, and resuspended with PBS containing 2% BSA. Annexin V-FITC Apoptosis Detection Kit (KeyGEN, ^#^KGA105) and Cell Cycle Detection Kit (KeyGEN, ^#^KGA511) were used for flow cytometry. For apoptosis staining, cells were mixed with Annexin V-FITC and propidium iodide (PI) reagents and were incubated for 10 min at room temperature in the dark. After fixation in 70% cold ethanol at 4°C for 12 h, PI containing RNase A was added into the resuspended cells for 45 min at room temperature in the dark for cell cycle assay. Flow cytometer (Beckman Coulter CytoFLEX, USA) was used to detect FITC and PI. Annexin V^+^ populations were considered as apoptotic cells, including PI^−^ early apoptotic cells and PI^+^ late apoptotic cells. The flow cytometry data were processed by FlowJo vX.0.7 (BD, USA) to evaluate apoptosis and cell cycle.

### 2.5. Western Blotting for Protein Expression

The total cell protein was extracted with cold RIPA lysis buffer containing phenylmethylsulfonyl fluoride (PMSF) (Whole Cell Lysis Assay Kit, KeyGEN, ^#^KGP250). The protein concentration of the extracts was determined by BCA Protein Quantitation Assay Kit (KeyGEN, ^#^KGP902) using a microplate reader. Then, SDS-PAGE loading buffer (KeyGEN, ^#^KGP101) was added to denature the protein at 100°C in thermostat. The prepared protein samples and prestained protein mass marker (KeyGEN, ^#^KGM441) were added to a 10% SDS-polyacrylamide gel with equal mass for electrophoresis (Bio-Rad Power Supplies Basic, USA). Then, the protein transfer system (Bio-Rad Trans-Blot Turbo, USA) was used to transfer the separated proteins from the gel to a polyvinylidene fluoride (PVDF) membrane, and 5% skim milk was used to eliminate nonspecific binding. Under the instruction of the prestained marker, the PVDF membranes were cut according to the protein mass and were incubated with primary antibodies overnight in a shaker at 4°C, including rabbit monoclonal to MGMT (Abcam, ^#^ab108630, dilution ratio 1 : 1000), rabbit monoclonal to P glycoprotein (MDR1, Abcam, ^#^ab168337, dilution ratio 1 : 1000), rabbit monoclonal to LXR*α* (Abcam, ^#^ab176323, dilution ratio 1 : 2000), and mouse monoclonal to GAPDH (Abcam, ^#^ab8245, dilution ratio 1 : 2000). After washing the PVDF membranes combined with primary antibodies, goat anti-rabbit IgG-HRP (for rabbit antibody, KeyGEN, ^#^KGAA35, dilution ratio 1 : 10000) or goat anti-mouse IgG-HRP (for mouse antibody, KeyGEN, ^#^KGAA37, dilution ratio 1 : 10000) was added to continue incubating. The electrochemiluminescence reagent (KeyGEN, ^#^KGP1121) was dripped on the PVDF membrane after antibody labeling, and a gel imaging system (Syngene G : BOX Chemi XR5, UK) was used to photograph the blots. The raw images of the immunoblot bands are presented in Supplementary Figure S2. The images were subjected to background removal and gray value (integrated density) determination via ImageJ 1.51j8 (National Institutes of Health, USA). The integrated density of (sample target blot/control target blot)/(sample GAPDH blot/control GAPDH blot) was considered as the relative expression of the target proteins.

### 2.6. Cholesterol Content Detection

Free cholesterol in the collected cells was removed by centrifugation. BCA Protein Quantitation Assay Kit (KeyGEN, ^#^KGP902) was used to determine protein concentration. Cells were treated with isopropanol and sonicated in the ice bath to prepare cell lysates. The total cholesterol content detection kit (Solarbio, ^#^BC1985) was used to detect the cholesterol concentration in the lysates. The samples, standard, and control solutions were treated with a reaction solution containing cholesterol ester hydrolase, cholesterol oxidase, and peroxidase at 37°C for 15 min, and OD values at 500 nm were measured with a microplate reader (MD SpectraMax M3, USA). After subtracting the OD value of the blank wells, the total cholesterol concentrations of the samples were calculated by the standard curve, and the total cholesterol concentration/protein concentration was calculated as the total cholesterol content.

### 2.7. Cholesterol Efflux Assay

For cholesterol tracking, 10 *μ*g/mL 25-NBD cholesterol (Sigma-Aldrich, ^#^810250P) was added to the cells in serum-free medium. After 24 h, the 25-NBD cholesterol that had not entered the cell was washed away. 6h before the end of drug treatment, 1 mM sodium taurocholate (NaTC, Solarbio, ^#^YZ-110815) was added as a receptor for ABCA1-mediated cholesterol efflux, which acts similarly to apolipoprotein A-I [35, 36]. After 6 h, the culture supernatant and pelleted cells were collected separately. Cells were lysed by 0.1 M NaOH for 30 min. The entire process was protected from light. A fluorescence microplate reader (BioTek ELx800, USA) was used to measure the fluorescence intensity (FI) at 469/537 nm of Abs/Em, and the cholesterol efflux rate was calculated. Cholesterol efflux rate = (FI_supernatant_/(FI_supernatant_ + FI_cell_)) × 100%.

### 2.8. Lipid Raft Labeling and Confocal Laser Scanning

After the cells were fixed with 4% formaldehyde for 15 min at 4°C, the Vybrant Alexa Fluor 594 Lipid Raft Labeling Kit (Invitrogen, ^#^V34405) was used to label lipid rafts by cholera toxin subunit B (CT-B). After incubating with Alexa Fluor 594-conjugated CT-B for 10 min at 4°C, the cells were centrifuged and resuspended in anti-CT-B antibody solution and kept at 4°C for 15 min in the dark. After lipid raft labeling, the cells were washed and treated with 4′, 6-diamidino-2-phenylindole (DAPI, KeyGEN, ^#^KGA215-50) for 10 min to stain the nucleus. After washing, cells were resuspended in cold PBS and photographed using a confocal laser scanning microscope (Olympus FV3000, Japan) at Ex/Em = 594/618 nm for Alexa Fluor 594 and Ex/Em = 359/461 nm for DAPI.

### 2.9. Quantitative Real-Time PCR (qRT-PCR) for mRNA Expression

Total cell RNA was extracted with TRIzol reagent (Invitrogen, ^#^15596–026). RNA concentration (OD260) and purity (OD260/280) were determined by UV spectrophotometer (Shimadzu UV-2450, Japan), and RNA with satisfactory purity (OD260/280 1.8–2.1) was quantified to 200 ng/*μ*l. RT Master Mix (Takara, ^#^RR036B) reagent and thermal cycler (ABI Veriti, USA) were used to reverse transcribe the RNA samples into cDNA. cDNA samples, primers, and PCR Master Mix containing SYBR Green (Takara, ^#^RR086B) were added to the PCR plate to detect the Ct values (amplification cycles) of *SREBF2*, *NR1H3*, *ABCA1,* and *GAPDH* mRNA through a qRT-PCR system (ABI StepOnePlus, USA). The relative expression of target mRNAs was calculated as 2^−ΔΔCt^ {ΔΔCt = (Ct (target RNA in sample well)−Ct (GAPDH in sample well))−(Ct (target RNA in control well)−Ct (GAPDH in control well))}. The primers (Table 1) were designed by KeyGEN BioTECH (China) using Primer 6.0 (Premier, Canada) and purified by PAGE. The amplification efficiency of primers was detected by cDNA standards prepared from U251 cells. The total RNA of U251 cells was quantified to 80 ng/*μ*l and mixed with a Total-Transcriptome cDNA Synthesis Reagent (ABM, ^#^G904) for reverse transcription by a thermal cycler (Bio-Rad C1000 Touch, USA), and five 10-fold concentration gradient cDNA standards were prepared. A qRT-PCR system (ABI QuantStudio 5, USA) was used to detect Ct values of cDNA standards after mixing with primers and BlasTaq 2X qPCR MasterMix Reagent (ABM, ^#^G891). Linear regression was executed according to the gene copies and Ct values of the standards, and the amplification efficiency (*E*) was calculated by the slope, *E*= (10^−1/slope^−1) × 100% (Table 1, Supplementary Figure S3).

### 2.10. Statistics and Graphs

GraphPad Prism 8.3.0 (GraphPad Software, USA) was used to perform statistical tests and data graphing. The RNA-seq data from CGGA were analyzed by the two-way ANOVA, Person correlation test, linear regression, and residual test. To calculate IC_50_, a variable slope nonlinear regression was performed. Other data were analyzed by the one-way ANOVA and passed the Brown–Forsythe test of variance homogeneity. *P* < 0.05 was considered statistically significant.

## 3. Results

### 3.1. Cholesterol Promoted the Resistance of GBM Cells to TMZ

We first analyzed the correlation between drug resistance and cholesterol metabolism through the CGGA database. By comparing the levels of cholesterol metabolism genes in high-grade glioma (HGG, WHO III-IV) tissues of patients receiving TMZ therapy with OS less than half a year and 5 years (Supplementary Figure S4(a)), we found that the level of *NR1H3* (transcribing LXR*α*) mRNA that mediates cholesterol efflux in patients with longer survival is higher than that with shorter survival. The clinical response of GBM patients to TMZ is related to the methylation status of MGMT and the expression of *MDR1* [6, 8], but the levels of cholesterol metabolism genes displayed a correlation with *MDR1* expression rather than MGMT methylation (Supplementary Figures S4(b) and S4(c)). *LDLR* that mediates cholesterol uptake and *SREBF2* and *HMGCR* that mediate cholesterol synthesis were positively correlated with the level of *MDR1*, and *NR1H3* that mediates cholesterol release was negatively correlated. These findings imply that cholesterol is related to MDR1-related TMZ resistance and the prognosis of GBM patients.

**Figure 2:**
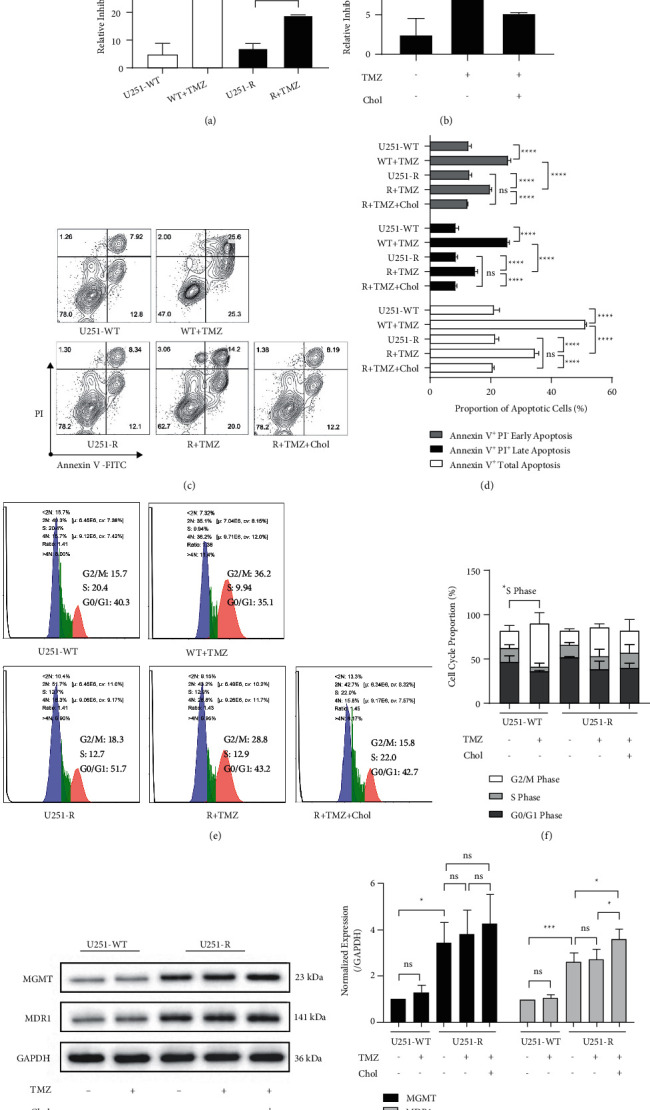
Cholesterol promoted temozolomide (TMZ) resistance of U251 cells. (a) Inhibition rate of 200 *μ*M TMZ or DMSO on U251 wild-type (U251-WT) and TMZ-resistant U251 (U251-R) cells evaluated by CCK-8. (b) Inhibition rate of 200 *μ*M TMZ on U251-R cells treated with 5 *μ*g/ml cholesterol (Chol) or DMSO. (c, d) The apoptosis proportion of U251-WT and U251-R cells treated with DMSO, 200 *μ*M TMZ alone, or combined with 5 *μ*g/ml Chol. (e, f) The cell cycle of U251-WT and U251-R cells treated with DMSO, 200 *μ*M TMZ alone, or combined with 5 *μ*g/ml Chol. (g, h) The protein expression of MGMT and MDR1 (integrated density referred to GAPDH) in DMSO, 200 *μ*M TMZ, or additional 5 *μ*g/ml Chol-treated U251-WT and U251-R cells. The histograms show the mean, and the error bars indicate SD. The percentage of cells (%) is displayed in the flow cytometry chart. *P*^*∗*^ < 0.05, *P*^*∗∗*^ < 0.01, *P*^*∗∗∗*^ < 0.001, *P*^*∗∗∗∗*^ < 0.0001; ns, no significant.

To explore whether cholesterol affects the TMZ resistance of GBM cells, we induced TMZ-resistant U251 GBM cells (resistance index = 8.784, Supplementary Figure S5 and Table S2). The levels of viable wild-type and resistant cells were similar, and TMZ inhibited the survival of both cell lineages, but the inhibition of TMZ on the resistant cells was considerably weaker than wild type (Figure 2(a)). The reaction of resistant strains to TMZ was further attenuated after cholesterol addition and was even comparable to that of the untreated resistant strains (Figure 2(b)). We further verified the protective effects of cholesterol on resistant U251 cells from TMZ pressure through flow cytometry. Compared with Annexin V^+^ apoptosis cells generally induced by TMZ in wild-type U251 cells (Figures 2(c) and 2(d)), including PI^−^ early apoptosis and PI^+^ late apoptosis, the level of apoptosis in resistant strains was moderate. The addition of cholesterol decreased apoptotic resistant cells in each phase, which was similar to untreated resistant cells. As a non-cell cycle-specific drug, TMZ displayed a slight effect on the cell cycle (Figures 2(e) and 2(f)). We only observed the shortening of S phase in wild-type cells, while cholesterol showed no further effect on the cell cycle. The above results demonstrated that cholesterol supports TMZ resistance of U251 cells.

As the most cholesterol-rich organ [16], the brain provides a unique microenvironment for GBM. GBM cells not only retain the ability of glial cells to synthesize cholesterol de novo but also increase cholesterol uptake mediated by low-density lipoprotein receptor (LDLR) [17]. Stimulating the liver X receptor (LXR) to promote ABCA1-mediated cholesterol efflux and degradation of LDLR induced GBM cell death [18–20]. It seems that the enormous demand for cholesterol from GBM cells is not just for the production of organelles. The increase in cholesterol and LDL levels promotes resistance of various tumors to chemotherapy, and it is also an independent risk factor for poor prognosis of GBM under standard TMZ treatment [21, 22]. Although cholesterol-induced drug resistance has not been confirmed in CNS tumors, elevated cholesterol in malignant ascites was proved to induce chemotherapy resistance by upregulating the drug efflux proteins ABCG1 and MDR1 [23]. MDR1 is located and stably expressed in lipid rafts composed of cholesterol, sphingolipid, and proteins on the plasma membrane [24]. When lipid rafts were induced to accumulate in specific parts of the membrane, MDR1 expression was confined, and tumor cells were less resistant [25]. Moreover, additionally added cholesterol restored the homogenous (broad and low-density) distribution of lipid rafts and upregulated MDR1 [25]. Conversely, lipid rafts were depleted when cholesterol was scavenged, and chemotherapy resistance was correspondingly diminished [26].

Both ginsenosides and cholesterol have steroid core structures (Figure 1), and ginsenosides can regulate cholesterol metabolism in tumor cells. Rg3 and CK hindered cholesterol synthesis by inhibiting *β*-hydroxy-*β*-methylglutaryl coenzyme A (HMG-CoA) [27, 28], and CK also promoted cholesterol efflux by stimulating LXR*α* [29, 30] to synergistically decrease intracellular cholesterol. Rh2 revealed lipid raft depleting activity [31, 32]. More direct evidence indicates that the ginsenoside derivative Rp1 decreased MDR1 activity by redistributing lipid rafts and attenuated the cholesterol-dependent resistance [25]. Consequently, ginsenosides are potential agents for the treatment of tumor resistance as regulators of cellular cholesterol metabolism. However, the differential roles of ginsenoside subtypes that govern cholesterol metabolism and drug resistance in CNS tumors remain undisclosed. In this study, we certified the role of cholesterol in TMZ resistance of glioblastoma U251 cells and investigated the effects of several ginsenosides with BBB penetration on TMZ resistance of U251 cells in the presence of cholesterol. We further studied the roles of ginsenosides on cholesterol metabolism and lipid raft distribution and the mechanisms controlling TMZ resistance. Our work suggests that ginsenosides Rg1 and CK are potential drugs to control TMZ resistance, providing references for adjuvant therapy of GBM.

### 3.2. Cholesterol Metabolism and Lipid Raft Distribution in TMZ-Resistant GBM Cells Were Significantly Different from Wild Type

**Figure 3:**
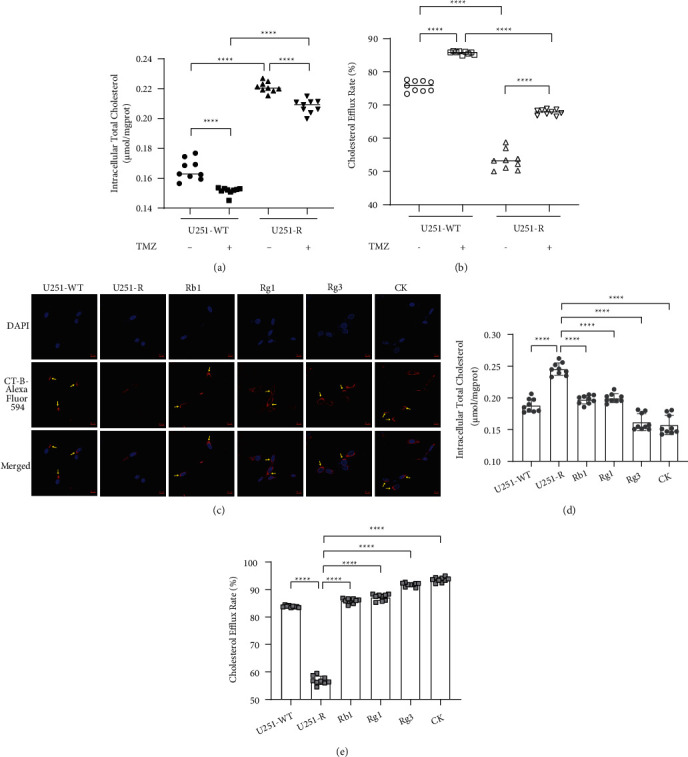
Ginsenosides Rb1, Rg1, Rg3, and compound K (CK) regulated cholesterol accumulation, efflux, and lipid raft distribution in temozolomide (TMZ)-resistant U251 (U251-R) cells. (a) Intracellular cholesterol concentrations of U251 wild-type (U251-WT) and resistant cells treated with 200 *μ*M TMZ or DMSO. (b) NaTC-induced 25-NBD cholesterol (10 *μ*g/mL) efflux rate of U251-WT and U251-R cells treated with 200 *μ*M TMZ or DMSO. (c) Intracellular cholesterol concentration of U251-WT cells treated with DMSO and U251-R cells treated with 100 *μ*M ginsenoside or DMSO. All cells were pretreated with 5 *μ*g/ml cholesterol. (d) NaTC-induced cholesterol efflux rate in U251-WT and U251-R cells. The cells were incubated with 10 *μ*g/mL 25-NBD cholesterol and treated with DMSO or 100 *μ*M ginsenosides. (e) Confocal laser scanning of the distribution of lipid rafts labeled with cholera toxin subunit B (CT-B) in U251-WT and U251-R cells treated with 5 *μ*g/ml cholesterol and 100 *μ*M ginsenoside or DMSO. Alexa Fluor 594-conjugated CT-B emits red fluorescence, and the blue fluorescence shows 4′, 6-diamidino-2-phenylindole (DAPI)-labeled nucleus. The arrows point to the area where lipid rafts accumulated on the cell membrane (magnification, × 600; scale bar, 10 *μ*m). The dots represent the value of each sample, the midlines or histograms display the mean, and the error bars indicate SD. *P*^*∗∗∗∗*^ < 0.0001.

As cholesterol attenuated the cytotoxicity of TMZ to resistant U251 cells and increased the expression of MDR1, we studied the cholesterol metabolism characteristics of TMZ-resistant U251 cells, to assess the potential of resistant cells to utilize cholesterol. When exposed to cholesterol, TMZ-resistant U251 cells preserved higher levels of intracellular cholesterol than wild type (Figure 3(a)), which may be due to cytotoxicity-mediated disruption of membrane integrity. We subsequently treated two cell lines with 25-NBD cholesterol to assay the cholesterol efflux provoked by the extracellular receptor NaTC [35, 36]. We found that the cholesterol efflux level of resistant U251 cells was markedly lower than wild type (Figure 3(b)). In addition, TMZ slightly reduced the content of intracellular cholesterol in both wild-type and resistant cells and promoted cholesterol efflux, implying that TMZ has a weak activity on cholesterol metabolism, which may also be related to its cytotoxic effect. The results reveal that TMZ-resistant GBM cells tend to retain more cholesterol and are associated with their resistance to chemotherapy.

MDR1 expression positively correlated with the homogenous distribution (broad and low density) of lipid rafts, which was attenuated upon the accumulation of lipid rafts and restored to a homogenous distribution after cholesterol uptake [25]. To confirm this phenomenon in GBM cells, we used fluorescently coupled CT-B to label lipid rafts [37, 38]. Under laser confocal microscopy, the CT-B signal of wild-type U251 cells was concentrated in a small area of the plasma membrane, while CT-B bound to TMZ-resistant strains tended to be distributed widely, homogenously, and moderately (Figure 3(c)).

### 3.3. Ginsenosides Attenuated Cholesterol Accumulation and Redistributed Lipid Rafts in TMZ-Resistant GBM Cells

Since the heterogeneous cholesterol metabolism properties of TMZ-resistant U251 cells, we studied the effects of brain tissue-permeable ginsenosides Rb1, Rg1, Rg3, and CK [12–14, 39] on cholesterol accumulation and efflux. All four ginsenosides decreased cholesterol content in resistant U251 cells (Figure 3(d)) and promoted NaTC-mediated cholesterol efflux (Figure 3(e)). Rg3 and CK exhibited the most pronounced effects.

Next, we studied the impact of ginsenosides on the distribution of lipid rafts. We found that the CT-B-binding lipid raft areas of the ginsenoside-treated TMZ-resistant strains exhibited different degrees of redistribution (Figure 3(c)). Among the resistant U251 cells, lipid rafts were significantly concentrated in partial areas after Rb1 and CK treatment, while the CT-B-labeled regions of the cells treated with Rg1 and Rg3 increased in density only in part of the plasma membrane, but were still widely distributed. Consequently, ginsenosides Rb1, Rg1, Rg3, and CK can redistribute lipid rafts, which are potentially associated with a decline in MDR1 expression. Among them, Rb1 and CK can redistribute lipid rafts more markedly.

### 3.4. Ginsenosides Increased LXR*α* Expression in TMZ-Resistant GBM Cells

**Figure 4:**
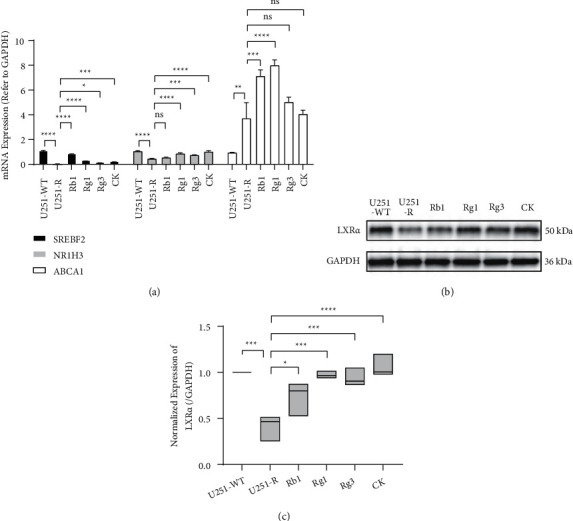
Ginsenosides modulated the transcription and translation of cholesterol metabolism factors in temozolomide (TMZ)-resistant U251 (U251-R) cells. (a) The expression of *SREBF2*, *NR1H3*, and *ABCA1* mRNAs in wild-type U251 (U251-WT) and U251-R cells. All cells were pretreated with 5 *μ*g/ml cholesterol. (b, c) The protein expression of LXR*α* and ABCA1 (integrated density referred to GAPDH) in U251-WT and U251-R cells. U251-WT cells were treated with DMSO, and U251-R cells were treated with 100 *μ*M Rb1, Rg1, Rg3, compound K (CK), or DMSO. The top of the histograms or the center lines of boxes display the mean, and the error bars indicate SD. *P*^*∗*^ < 0.05, *P*^*∗*^ < 0.05, *P*^*∗∗*^ < 0.01, *P*^*∗∗∗*^ < 0.001, *P*^*∗∗∗∗*^ < 0.0001; ns, no significant.

To study how ginsenosides regulate cholesterol aggregation and efflux, we assayed the expression of metabolism genes, including *NR1H3* (LXR*α*) that activates cholesterol efflux, cholesterol efflux transporter *ABCA1*, and *SREBF2*, which mediates the de novo synthesis of cholesterol. Compared with wild-type U251 cells, the transcription of *NR1H3* considerably diminished in TMZ-resistant cells, but ginsenosides Rg1, Rg3, and CK reversed this phenomenon to varying degrees (Figure 4(a)). Unexpectedly, the *ABCA1* transcription in the TMZ-resistant U251 cells was higher than the wild type. Although Rb1 displayed no effect on *NR1H3* mRNA, Rb1 and Rg1 both further upregulated *ABCA1* mRNA in resistant cells. In addition, compared with the wild-type cells, the expression of *SREBF2* mRNA in TMZ-resistant strains was almost undetectable. The expression of *SREBF2* mRNA in resistant cells treated with these ginsenosides was restored to a certain extent, but the upregulation mediated by Rg1, Rg3, and CK was subdued.

Due to the gene expression results, the effects of ginsenosides on intracellular cholesterol content may depend on cholesterol efflux mediated by LXR*α*, rather than attenuating SREBF2-mediated cholesterol synthesis. To verify this conjecture, we next identified the expression of LXR*α* on the protein level and found the reduced expression of LXR*α* in TMZ-resistant U251 cells (Figures 4(b) and 4(c)). Similarly, ginsenosides Rb1, Rg1, Rg3, and CK upregulated the protein expression of LXR*α*, particularly Rg1 and CK. The results disclose that ginsenosides Rb1, Rg1, Rg3, and CK may decrease the intracellular cholesterol content and promote cholesterol efflux in TMZ-resistant GBM cells by upregulating LXR*α*.

### 3.5. Ginsenosides Rg1 and CK Improved Cholesterol Efflux and Inhibited TMZ Resistance in GBM Cells by Upregulating LXR*α*

**Figure 5:**
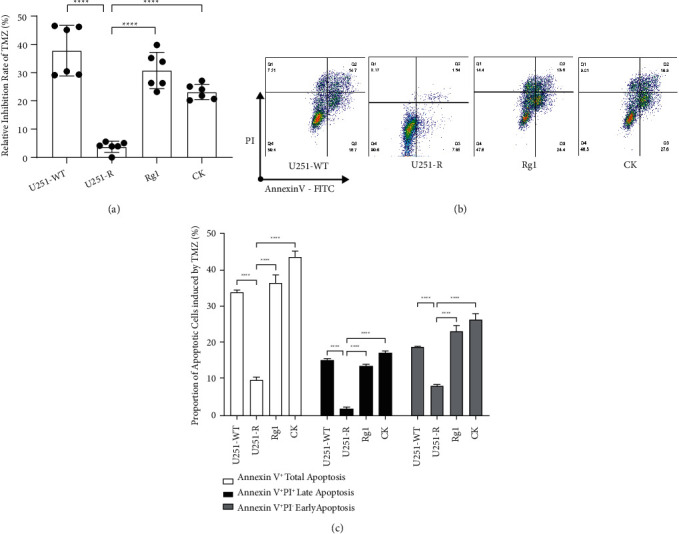
Ginsenosides Rg1 and compound K (CK) raised the sensitivity of resistant U251 cells (U251-R) to temozolomide (TMZ) in the presence of cholesterol. (a) Inhibition rate on U251 wild-type (U251-WT) and U251-R cells evaluated by CCK-8. (b, c) Proportion of apoptosis in U251-WT and U251-R cells at each stage. After incubation with 5 *μ*g/ml cholesterol, U251-WT cells were treated with DMSO, and U251-R cells were treated with DMSO, Rg1, or CK. *P*^*∗∗∗∗*^ < 0.0001.

Given that Rg1 and CK showed better upregulation of LXR*α* among the four ginsenoside subtypes (Figures 4(b) and 4(c)), we consider that these two ginsenosides have more potential to control the TMZ resistance of GBM cells in the presence of cholesterol. The results indicated that resistant U251 cells were more resistant to TMZ stress than wild type in the presence of cholesterol. The cells were nearly uninhibited (Figure 5(a)), and the level of apoptosis was lower at all stages, especially at the Annexin V^+^ PI^+^ late stage (Figures 5(b) and 5(c)). The additional treatment of Rg1 and CK restored the sensitivity of resistant cells to TMZ and promoted TMZ-induced cell apoptosis at all stages. To explore how ginsenosides Rg1 and CK attenuated TMZ resistance in GBM cells in cholesterol-containing matrices, we focused on LXR*α*.

**Figure 6:**
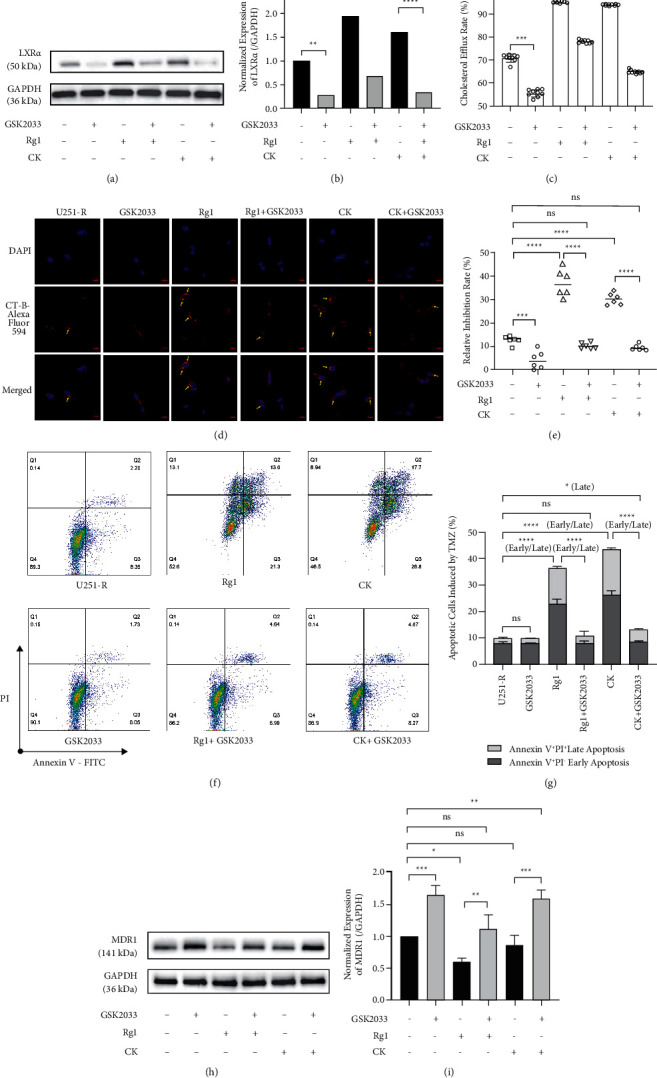
Ginsenosides Rg1 and compound K (CK) regulated cholesterol metabolism and lipid raft distribution and inhibited the temozolomide (TMZ) resistance of U251 cells by upregulating LXR*α*. (a, b) The protein expression of LXR*α* (integrated density referred to GAPDH) in TMZ-resistant U251 (U251-R) cells. (c) NaTC-induced cholesterol efflux rate in U251-R cells incubated with 10 *μ*g/mL 25-NBD cholesterol. (d) Confocal laser scanning of the distribution of lipid rafts labeled with cholera toxin subunit B (CT-B) in U251-R cells. Alexa Fluor 594-conjugated CT-B emits red fluorescence, and the blue fluorescence shows 4′, 6-diamidino-2-phenylindole (DAPI)-labeled nucleus. The arrows point to the area where lipid rafts accumulated on the cell membrane (magnification, ×600; scale bar, 10 *μ*m). (e) Inhibition rate on U251-R cells assessed by CCK-8. (f, g) The proportion of apoptotic U251-R cells at each stage. (h, i) The protein expression of MDR1 (integrated density referred to GAPDH) in U251-R cells. Cells in all groups were pretreated with 5 *μ*g/ml cholesterol and dosed with 200 *μ*M TMZ, and cells were additionally supplemented with 100 *μ*M Rg1 or CK in the presence or absence of 100 nM GSK2033. The histogram and dots represent the mean, and the error bars indicate SD. *P*^*∗*^ < 0.05, *P*^*∗*^ < 0.05, *P*^*∗∗*^ < 0.01, *P*^*∗∗∗*^ < 0.001, *P*^*∗∗∗∗*^ < 0.0001; ns, no significant.

In consideration of the correlation of LXR*α* with the survival and transcription level of MDR1 in TMZ-treated HGG patients (Supplementary Figure S4), we evaluated the role of LXR inhibitor GSK2033 on the effects of ginsenosides Rg1 and CK in regulating cholesterol metabolism and TMZ resistance. GSK2033 greatly inhibited LXR*α* expression in TMZ-resistant U251 cells and blocked the induction of LXR*α* translation by Rg1 and CK (Figures 6(a) and 6(b)). Following changes in LXR*α* levels, GSK2033 inhibited cholesterol efflux to NaTC in resistant cells and blocked the facilitation of cholesterol efflux by Rg1 and CK (Figure 6(c)). GSK2033 also more homogenously distributed lipid rafts in resistant U251 cells and reversed the aggregated remodeling of lipid rafts by Rg1 and CK (Figure 6(d)). These changes in cholesterol metabolism were ultimately reflected in the cytotoxicity of TMZ to resistant GBM cells. In the presence of cholesterol, GSK2033-mediated LXR*α* inhibition resisted the sensitization of Rg1 and CK to TMZ (Figures 6(e), 6(f), and 6(g)). It is worth mentioning that even in the presence of GSK2033, CK still increased the level of Annexin V^+^ PI^+^ late apoptosis in resistant U251 cells. Besides, GSK2033 increased MDR1 expression in resistant U251 cells exposed to cholesterol while regulating lipid raft distribution (Figures 6 (h)and 6(i)). Although Rg1 downregulated MDR1 expression in resistant cells, this effect was inhibited by GSK2033. No effect of CK on MDR1 expression was identified, although CK increased the cytotoxicity of TMZ to resistant cells. Our work not only identified ginsenosides Rg1 and CK with activity to attenuate TMZ resistance but also confirmed that the cytotoxic sensitization of these ginsenosides was through stimulating LXR*α*-mediated cholesterol efflux and lipid raft redistribution.

## 4. Discussion

Tumor cells are adaptable to the environment. In addition to microenvironmental oxygen, glucose, and glutamine, cholesterol has also become an essential material basis for tumor cells to attain energy and synthesize organelles after metabolic reprogramming [40]. Although astrocytes are the main source of cholesterol in brain tissues [41], cholesterol from peripheral blood can also be taken up through the BBB expressing abundant LDLR [42]. Hence, the brain tumor microenvironment possesses high levels of cholesterol that can be deployed by GBM cells. We identified that TMZ resistance of GBM cells was dependent on cholesterol in the microenvironment. The addition of cholesterol led to attenuated apoptosis and reduced expression of the lipid raft-related drug resistance protein MDR1 in resistant cells, which tended to retain more cholesterol intracellularly with inhibited efflux and maintained lipid raft distribution. MGMT, which mediates the alkylation repair of the 6^th^ oxygen of guanine on DNA, is another TMZ resistance vector of GBM cells. Both hypermethylation status of MGMT promoter and MGMT overexpression are independent risk factors for desensitization to TMZ therapy [8, 43]. Although the increased expression of MGMT was observed in the resistant cells, the addition of cholesterol failed to further modify its level. Different from MGMT, the expression and activity of MDR1 are determined by lipid rafts and are associated with cholesterol-maintained lipid raft homeostasis [24, 25]. Consequently, faded cholesterol efflux and stable lipid raft distribution may be involved in sustaining the expression of MDR1 in resistant GBM cells. The development of drugs to target cholesterol metabolism is a favorable strategy to control TMZ resistance in GBM.

Ginsenosides are the active components of the *Panax genus*, *Araliaceae*, which have a steroid structure similar to cholesterol and have the potential to govern cholesterol metabolism. Rb1, Rg1, Rg3, and CK are subtypes of ginsenosides that have been reported to have CNS effects and brain penetration [12–14, 39]. Rb1, Rg3, and CK belong to protopanaxadiol, while Rg1 belongs to protopanaxatriol. We found that Rb1, Rg1, Rg3, and CK induced cholesterol efflux in TMZ-resistant GBM cells, reduced intracellular cholesterol concentrations, and redistributed lipid rafts at different levels. It has been reported that CK promoted cholesterol efflux by stimulating LXR*α* [29, 30], and we recognized the increased expression of LXR*α* in resistant GBM cells after treated with Rb1, Rg1, and Rg3, in addition to CK. We further observed that Rg1 and CK raised the sensitivity of resistant GBM cells to TMZ, and this effect was via upregulation of LXR*α*. According to the structure of Rg1 and CK (Figure 1), hydroxyl at R1 position and O-*β*-D-glucopyranosyl at R3 position on the core of saponin may be effector groups stimulating LXR*α* and controlling drug resistance in GBM cells.

The increase in intracellular cholesterol level mediated by downregulation of LXR*α* plays an important role in promoting TMZ resistance in GBM cells. Based on our analysis of the CGGA database, in tumor tissues of GBM patients treated with TMZ, the level of LXR*α* mRNA (*NR1H3*) was associated with prolonged survival, while the level of the raft resistance gene MDR1 was negatively correlated with *NR1H3* and positively correlated with cholesterol uptake gene *LDLR* and synthetic gene *SREBF2*. It was recently reported that cholesterol accumulation mediated by the downregulation of LXR*α* promoted GBM cell growth, whereas the activation of LXR*α*-mediated cholesterol efflux exhibited the opposite effects [19, 44]. Although a study identified an increase in TMZ-induced apoptosis in resistant GBM cells treated with cholesterol [45], the dose of cholesterol was up to 20 *μ*g/ml [46]. We applied a small dose of cholesterol (5 *μ*g/ml) and found that cholesterol strengthened the TMZ resistance of GBM cells, and the expression of LXR*α* was depressed in resistant cells. In cholesterol-maintained TMZ-resistant cells, ginsenosides Rg1, Rg3, and CK upregulated LXR*α* and stimulated LXR*α*-mediated cholesterol efflux, which were initially depressed to retain intracellular cholesterol and improve lipid raft distribution. We demonstrated that subtypes Rg1 and CK increased the sensitivity of resistant cells to TMZ through their stimulatory effects on LXR*α* by a LXR inhibitor, further supporting the positive effect of this protein and cholesterol efflux in controlling resistance. The upregulation of LXR*α* and promotion of cholesterol efflux by CK were weaker than that of Rg1, which is consistent with the redistribution of lipid rafts. This may be the reason for the mild inhibitory effect of CK on the raft resistance protein MDR1. Nonetheless, from the results, CK and Rg1 displayed similar chemosensitization effects on resistant cells, implying that they are both potential agents for the treatment of TMZ resistance.

The drug resistance of GBM cells requires cholesterol, and GBM cells adjust their cholesterol homeostasis according to the concentration of environmental cholesterol. Studies have discovered that in the absence of external cholesterol, the intracellular cholesterol content of resistant U251 cells was higher than wild type [45], reflecting the activated cholesterol de novo synthesis. However, we found that the expression of the cholesterol synthesis gene *SREBF2* in TMZ-resistant GBM cells declined, and the cholesterol efflux was diminished after obtaining external cholesterol. The transcription level of *SREBF2* raised along with LXR*α* after treated with ginsenoside, indicating that GBM cells may ensure the intracellular supply of cholesterol by modifying the ability of cholesterol synthesis. Even so, the feedback upregulation of *SREBF2* is still less obvious than the upregulation of ginsenosides on LXR*α*, which ultimately performed as the inhibition of TMZ resistance. In view of the self-regulation of cholesterol metabolism in GBM cells, the development of cholesterol efflux stimulators such as ginsenosides Rg1 and CK, combined with the strategy of cholesterol synthesis inhibition, could block the TMZ resistance of GBM cells far more effectively than simply blocking cholesterol intake.

Although we demonstrated that ginsenosides Rg1 and CK controlled the resistance to TMZ in GBM cells by stimulating LXR*α*-mediated cholesterol efflux, some limitations remain in this study. The mechanism of resistance inhibition was initially confirmed by LXR inhibitors, which require further validation in LXR*α* knockout and overexpressing cells. What is more, to increase the feasibility of these ginsenosides in clinical studies, animal studies are required for data on *in vivo* efficacy against TMZ resistance and the concentration of each subtype in the cerebrospinal fluid after peripheral administration. These will be augmented in subsequent work.

## 5. Conclusions

**Figure 7:**
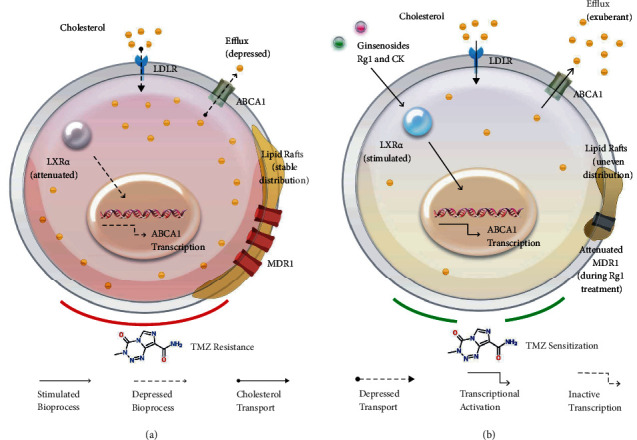
Ginsenosides Rg1 and compound K (CK) increased the sensitivity of resistant glioblastoma cells to temozolomide (TMZ) by governing cholesterol metabolism and lipid raft distribution. (a) Cholesterol in the brain tumor microenvironment is taken up in the form of cholesterol esters by low-density lipoprotein receptor (LDLR). Restricted expression of LXR*α* in TMZ-resistant glioblastoma cells leads to attenuated ABCA1-mediated cholesterol efflux, which retains a higher concentration of intracellular cholesterol to maintain a stable distribution of lipid rafts and increases the resistance to TMZ. The amplified TMZ resistance is potentially associated with the upregulation of MDR1 in lipid rafts. (b) Ginsenosides Rg1 and CK facilitated cholesterol efflux by stimulating the expression of LXR*α*. The decreased intracellular cholesterol redistributed lipid rafts in an uneven and aggregated state and reserved the cytotoxicity of TMZ on glioblastoma cells. Following the regulation of cholesterol metabolism, Rg1 treatment also diminished MDR1 expression.

Ginsenosides Rg1 and CK can induce cholesterol efflux by upregulating LXR*α*, decreasing intracellular cholesterol content, and redistributing lipid rafts in TMZ-resistant GBM cells. These modulations of cholesterol metabolism controlled the resistance of GBM cells to TMZ (Figure 7). Our findings uncovered a potential adjuvant drug for standard TMZ treatment, promising a novel approach for improving prognosis in GBM patients resistant to chemotherapy.

## Figures and Tables

**Table 1:** Primer parameters for qRT-PCR.

Gene	NCBI ID	Forward primer	Reverse primer	Efficiency (%)
*SREBF2*	6721	5′-TTGTCGGGTGTCATGGGC-3′	5′-ACAAATTGCAGCATCTCGTCG-3′	108.88
*NR1H3*	10062	5′-TCTGGACAGGAAACTGCACC-3′	5′-ACATCTCTTCCTGGAGCCCT-3′	113.12
*ABCA1*	19	5′-GGGTCTGTCCCCAGCATAAC-3′	5′-TCTGCATTCCACCTGACAGC-3′	101.44
*GAPDH*	2597	5′-AGATCATCAGCAATGCCTCCT-3′	5′-TGAGTCCTTCCACGATACCAA-3′	110.17

## Data Availability

RNA sequencing datasets generated and analyzed during this study are available in the Chinese Glioma Genome Atlas (CGGA) repository, https://www.cgga.org.cn/. Other data that support this study are available from the corresponding author on reasonable request.

## Abbreviations

BBB: Blood-brain barrier
CGGA: Chinese Glioma Genome Atlas
CK: Compound K
GBM: Glioblastoma
HGG: High-grade glioma
LDLR: Low-density lipoprotein receptor
MDR: Multidrug resistance protein
MGMT: O6-methylguanine-DNA methyltransferase
NaTC: Sodium taurocholate
PI: Propidium iodide
STR: Short tandem repeat
TMZ: Temozolomide.

## Supplementary Materials

Supplementary Materials The methods and results of STR identification of TMZ-resistant U251 cells, the amplification efficiency of primers, the analysis results of the transcriptome sequencing database, the validation of drug resistance, and the raw images of the immunoblot bands are presented in the supplementary materials. Figure S1. Genotyping map of short tandem repeat (STR) and Amelogenin locus in temozolomide-resistant U251 (U251TMZ) cells. About 1 × 10^6^ constructed temozolomide-resistant U251 cells were collected, and genomic DNA was extracted by PureLink Genomic DNA Mini Kit (Life Technologies, ^#^K182001). DNA was amplified by PowerPlex 18D System (Promega, ^#^DC1802), and the alleles were detected on STR and Amelogenin locus by an ABI 3500 Genetic Analyzer (Life Technologies, USA). The genetic test results of resistant cells were compared with cell line data in several databases to identify the genetic stability. The strain matching the resistant cells was named U251 MG (No. CVCL 0021 Best) in the EXPASY database (https://www.expasy.org/). No multiple alleles were detected in resistant cells. Figure S2. Integral images of the immunoblot bands used for analysis in the main text. 3 biological replicates were executed in each group. (a) The protein expression of MGMT, MDR1, and GAPDH during treatment of temozolomide (TMZ) and cholesterol (Chol). Immunoblot bands for MDR1 and GAPDH were cut from the same PVDF membrane, while MGMT was cut from the other. (b) The protein expression of LXR*α*, ABCA1, and GAPDH during treatment of ginsenosides in the presence of cholesterol. The immunoblot bands were cut from the same PVDF membrane. (c) The protein expression of LXR*α*, MDR1, and GAPDH during treatment of Rg1 and CK in the presence or absence of GSK2033 in cholesterol-containing medium. The immunoblot bands were cut from the same PVDF membrane. U251-WT, wild-type U251 cells, U251-R, and TMZ-resistant U251 cells. Figure S3. Amplification efficiency of primers for qRT-PCR, including *SREBF2*, *NR1H3*, *ABCA1,* and *GAPDH*. The *X*-axis represents the gene copies of the cDNA standards for 5 concentration gradients, and the *Y*-axis exhibits the amplification cycles (CT value) for each concentration. Figure S4. Correlation between temozolomide resistance and cholesterol metabolism genes including *LDLR*, *SREBF*, *HMGCR*, *NR1H3*, *ABCA1,* and *ABCG1* in adult patients with high-grade glioma through Chinese Glioma Genome Atlas (CGGA) database. (a) The difference in the expression of cholesterol metabolism mRNAs in tumor tissues between patients who received temozolomide treatment with OS < 0.5 years and OS ≥ 5 years. (b) The influence of the methylation status of MGMT on the expression of cholesterol metabolism mRNAs in tumor tissues. (c) The Pearson correlation test of the expression of resistance gene *MDR1* and cholesterol metabolism genes in tumor tissues. The mean lines are presented in the graph, and the error bars on both sides of the mean line indicate SD. *P*^*∗∗∗∗*^ < 0.0001; ns, no significant. Figure S5. Dose-viability curves of wild-type U251 (U251-WT) and temozolomide (TMZ)-resistant U251 (U251-R) cells treated with TMZ. The dots indicate the mean value of cell viability at each dose, and the error bars indicate SD.
