# Supplementary material for: Ginsenosides Rg1 and CK Control Temozolomide Resistance in Glioblastoma Cells by Modulating Cholesterol Efflux and Lipid Raft Distribution
Source: Evid Based Complement Alternat Med. 2022 Oct 10;2022:1897508. doi: 10.1155/2022/1897508 (PMC9583863; doi:10.1155/2022/1897508)
Supplement: Supplementary Materials — The methods and results of STR identification of TMZ-resistant U251 cells, the amplification efficiency of primers, the analysis results of the transcriptome sequencing database, the validation of drug resistance, and the raw images of the immunoblot bands are presented in the supplementary materials. Figure S1. Genotyping map of short tandem repeat (STR) and Amelogenin locus in temozolomide-resistant U251 (U251TMZ) cells. About 1 × 10^6^ constructed temozolomide-resistant U251 cells were collected, and genomic DNA was extracted by PureLink Genomic DNA Mini Kit (Life Technologies, #K182001). DNA was amplified by PowerPlex 18D System (Promega, #DC1802), and the alleles were detected on STR and Amelogenin locus by an ABI 3500 Genetic Analyzer (Life Technologies, USA). The genetic test results of resistant cells were compared with cell line data in several databases to identify the genetic stability. The strain matching the resistant cells was named U251 MG (No. CVCL 0021 Best) in the EXPASY database (https://www.expasy.org/). No multiple alleles were detected in resistant cells. Figure S2. Integral images of the immunoblot bands used for analysis in the main text. 3 biological replicates were executed in each group. (a) The protein expression of MGMT, MDR1, and GAPDH during treatment of temozolomide (TMZ) and cholesterol (Chol). Immunoblot bands for MDR1 and GAPDH were cut from the same PVDF membrane, while MGMT was cut from the other. (b) The protein expression of LXRα, ABCA1, and GAPDH during treatment of ginsenosides in the presence of cholesterol. The immunoblot bands were cut from the same PVDF membrane. (c) The protein expression of LXRα, MDR1, and GAPDH during treatment of Rg1 and CK in the presence or absence of GSK2033 in cholesterol-containing medium. The immunoblot bands were cut from the same PVDF membrane. U251-WT, wild-type U251 cells, U251-R, and TMZ-resistant U251 cells. Figure S3. Amplification efficiency of primers for qRT-PCR, including SREBF2, NR1H3, [file 1897508.f1.pdf]

Supplementary Materials

- Evidence-Based Complementary and Alternative Medicine

Ginsenosides Rg1 and CK Control Temozolomide Resistance in Glioblastoma Cells by Modulating Cholesterol Efflux and Lipid Raft Distribution

Runze Qiu, Jingjing Zhang, Chun Ge, Yue Zhong, Suo Liu, Qingquan Li, Jianjun Zou, Yingbin Li*, Hongwei Fan*

* Correspondence:

Hongwei Fan, Department of Clinical Pharmacology Laboratory, Nanjing First Hospital, Nanjing Medical University, Nanjing 210006, China, fanhongwei178@njmu.edu.cn

Yingbin Li, Department of Neurosurgery, the Second Affiliated Hospital of Nanjing Medical University, Nanjing 210011, China, yingbinli65@sina.com

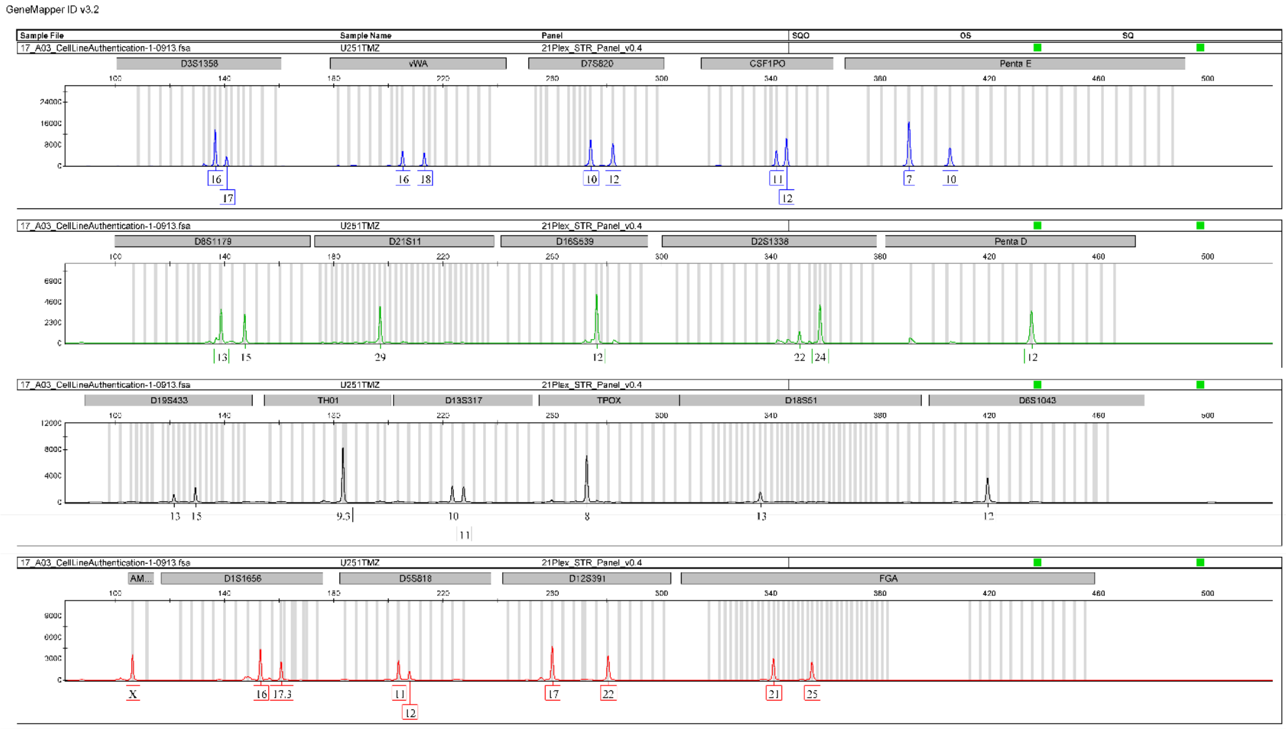

Figure S1: The genotyping map of short tandem repeat (STR) and Amelogenin loci in temozolomide-resistant U251 (U251TMZ) cells. About 1×10^6 constructed temozolomide-resistant U251 cells were collected, and genomic DNA was extracted by PureLink Genomic DNA Mini Kit (Life Technologies, #K182001). DNA was amplified by PowerPlex 18D system (Promega, #DC1802), and detected the alleles on STR and Amelogenin loci by an ABI 3500 Genetic Analyzer (Life Technologies, USA). The genetic test results of resistant cells were compared with cell line data in several databases to identify the genetic stability. The strain matching the resistant cells was named U-251 MG (No. CVCL 0021 Best) in the EXPASY database (https://www.expasy.org/). No multiple alleles were detected in resistant cells.

Table S1: Genotyping results of short tandem repeat (STR) and Amelogenin locis in temozolomide-resistant U251 (U251-R) cells compared to cell data in EXPASY database (https://www.expasy.org/).

Loci	Detected U251-R cells			Cell data in EXPASY (U-251 MG, #CVCL 0021 Best)		
	Allele1	Allele2	Allele3	Allele1	Allele2	Allele3
D5S818	11	12	/	11	12	/
D13S317	10	11	/	10	11	/
D7S820	10	12	/	10	12	/
D16S539	12	12	/	12	12	/
VWA	16	18	/	16	18	/
TH01	9.3	9.3	/	9.3	9.3	/
AMEL	X	X	/	X	X	/
TPOX	8	8	/	8	8	/
CSF1PO	11	12	/	11	12	/
D12S391	17	22	/	/	/	/
FGA	21	25	/	21	25	/
D2S1338	22	24	/	22	24	/
D21S11	29	29	/	/	/	/
D18S51	13	13	/	13	13	/
D8S1179	13	15	/	13	15	/
D3S1358	16	17	/	16	17	/
D6S1043	12	12	/	/	/	/
PENTAE	7	10	/	7	10	/
D19S433	13	15	/	13	15	/
PENTAD	12	12	/	12	12	/

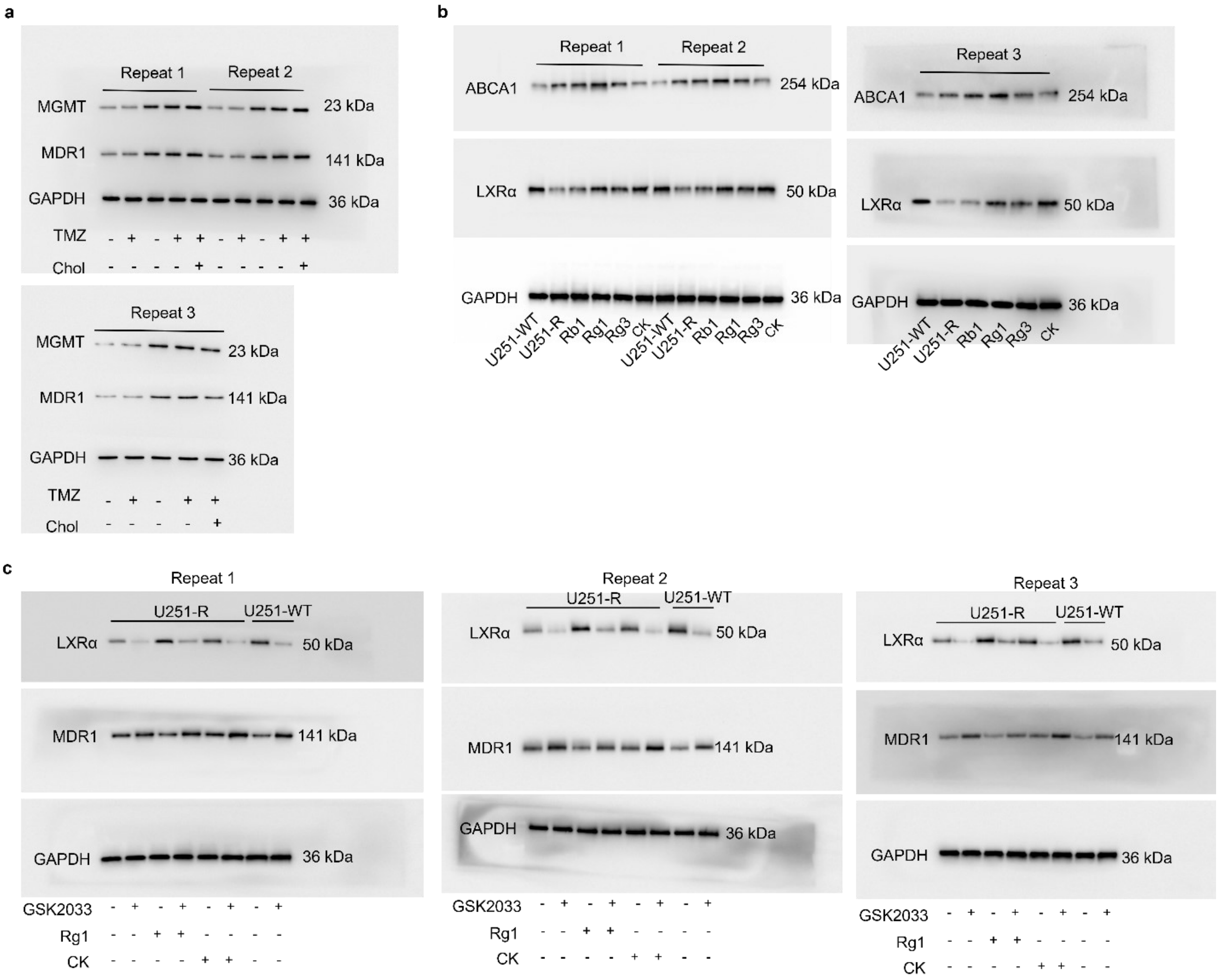

Figure S2: Integral images of the immunoblot bands used for analysis in the main text. 3 biological replicates were executed in each group. (a) The protein expression of MGMT, MDR1 and GAPDH during treatment of temozolomide (TMZ) and cholesterol (Chol). Immunoblot bands for MDR1 and GAPDH were cut from the same PVDF membrane, while MGMT was cut from the other. (b) The protein expression of LXRα, ABCA1 and GAPDH during treatment of ginsenosides in the presence of cholesterol. The immunoblot bands were cut from the same PVDF membrane. (c) The protein expression of LXRα, MDR1 and GAPDH during treatment of Rg1 and CK in the presence of absence of GSK2033 in cholesterol-containing medium. The immunoblot bands were cut from the same PVDF membrane. U251-WT, wild-type U251 cells, U251-R, TMZ-resistant U251 cells.

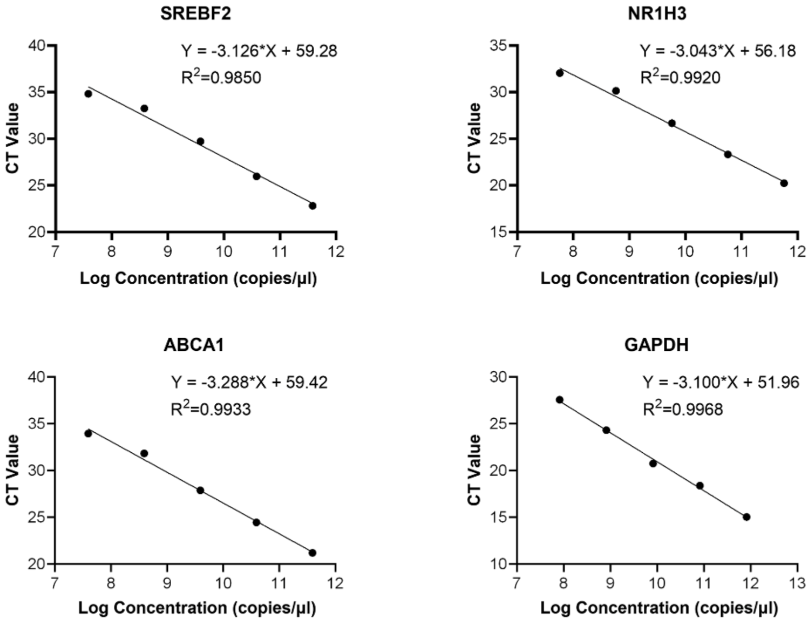

Figure S3: The amplification efficiency of primers for qRT-PCR, including *SREBF2*, *NR1H3*, *ABCA1* and *GAPDH*. The X-axis represents the gene copies of the cDNA standards for 5 concentration gradients, and the Y-axis exhibits the amplification cycles (CT Value) for each concentration.

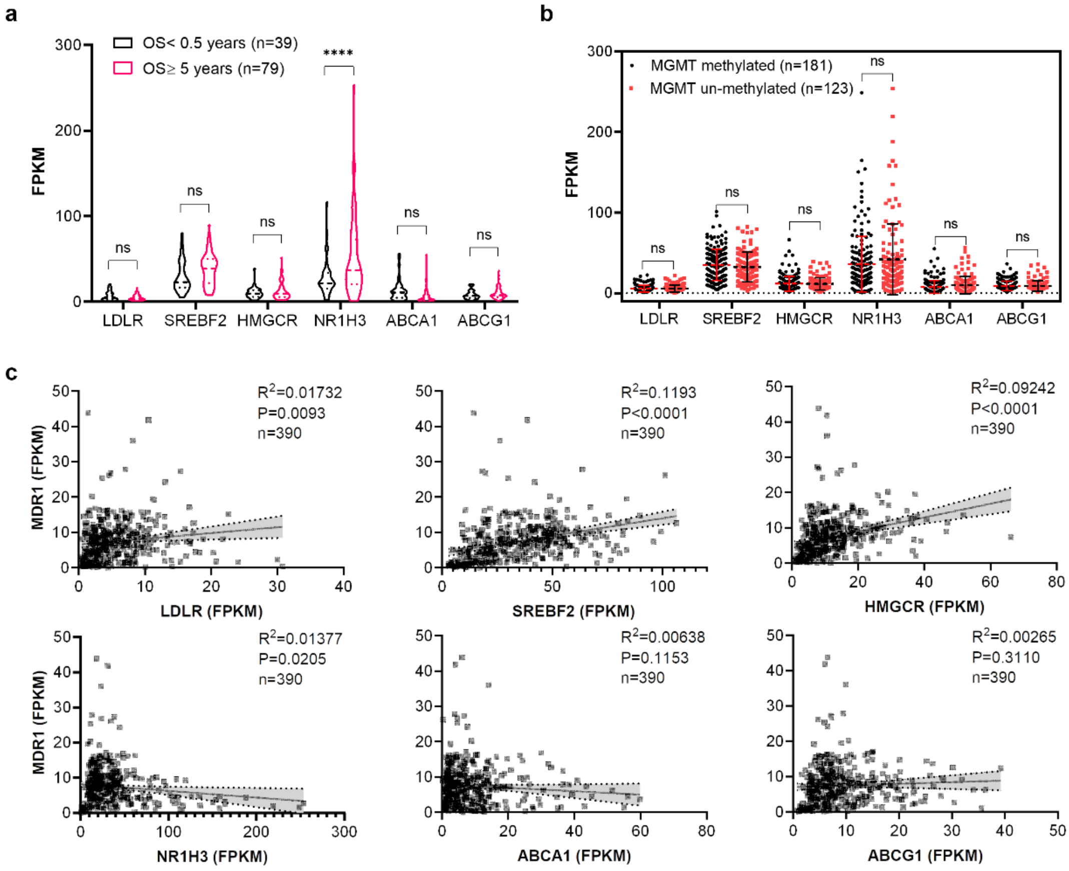

Figure S4: The correlation between temozolomide resistance and cholesterol metabolism genes including *LDLR*, *SREBF*, *HMGCR*, *NR1H3*, *ABCA1* and *ABCG1* in adult patients with high-grade glioma through Chinese Glioma Genome Atlas (CGGA) database. (a) The difference in the expression of cholesterol metabolism mRNAs in tumor tissues between patients who received temozolomide treatment with OS< 0.5 years and OS≥ 5 years. (b) The influence of the methylation status of MGMT on the expression of cholesterol metabolism mRNAs in tumor tissues. (c) The Pearson correlation test of the expression of resistance gene *MDR1* and cholesterol metabolism genes in tumor tissues. The mean lines are presented in the graph, and the error bars on both sides of the mean line indicate SD. $P^{****} < 0.0001$, ns, no significant.

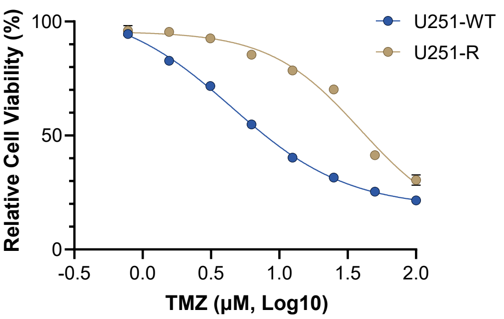

Figure S5: The dose-viability curves of wild-type U251 (U251-WT) and temozolomide (TMZ)-resistant U251 (U251-R) cells treated with TMZ. The dots indicate the mean value of cell viability at each dose, and the error bars indicate SD.

Table S2: The IC_{50} of TMZ to TMZ-resistant (U251-R) and wild-type U251 (U251-WT) cells, and the resistant index.

Cell	IC_{50} (μM)	Resistant Index
U251-WT	4.473	8.784
U251-R	39.29	
